# High Score of ELST‐Blue in Endoscopic Ultrasonography Strain Elastography May Provide a High Risk Group of Early Chronic Pancreatitis with the Reduction of Apolipoprotein A2‐i Index

**DOI:** 10.1002/deo2.70191

**Published:** 2025-08-29

**Authors:** Ken Nakamura, Seiji Futagami, Shuhei Agawa, Sakura Higashida, Tomohide Tanabe, Takeshi Onda, Rie Kawawa, Mayu Habiro, Kumiko Kirita, Songyu Sai, Norio Itokawa, Nobue Ueki, Yoshiyuki Watanabe, Ryo Ohta, Nobuhiko Taniai, Kazufumi Honda, Katsuhiko Iwakiri, Masanori Atsukawa

**Affiliations:** ^1^ Division of Gastroenterology Nippon Medical School Tokyo Japan; ^2^ Department of Internal Medicine Kawasaki Rinko General Hospital Kawasaki Japan; ^3^ Department of Gastrointestinal and Hepato‐Biliary‐Pancreatic Surgery Nippon Medical School Tokyo Japan; ^4^ Department of Bioregulation Graduate School of Medicine Nippon Medical School Tokyo Japan; ^5^ Institute of Advanced Medical Sciences Nippon Medical School Tokyo Japan

**Keywords:** apolipoprotein A2 | early chronic pancreatitis | elastography | endosonography | exocrine pancreatic function

## Abstract

**Background:**

To evaluate the usefulness of the ELST‐blue score to explore its potential application in identifying high‐risk groups for early chronic pancreatitis (ECP) through reflecting on pancreatic elasticity and the reduction of pancreatic function, and we tried to demonstrate whether the ELST‐blue score was significantly associated with apolipoprotein A2 (apoA2) isoforms in patients with ECP.

**Methods:**

Forty‐four patients with pancreatic enzyme abnormalities underwent endosonography. We divided two groups, one group was patients with ECP (*n* = 16) and the other group was patients with non‐ECP (*n* = 28). ELST‐blue was defined using the open‐source software ‘Image J’. The concentration of apoA2 isoforms was measured using an enzyme‐linked immunosorbent assay kit.

**Results:**

Epigastric pain tended to be more severe in patients with ECP than in those without ECP. There was a significant difference in the diameter of the main pancreatic duct of more than 2 mm as well as in stranding or hyperechoic foci and lobularity between patients with ECP and non‐ECP. The ELST‐blue score was significantly higher in patients with ECP than in non‐ECP (*p* = 0.003). Although an intense negative correlation was determined between ELST‐blue score and the apoA2‐i Index in patients with ECP (r = ‐0.704, *p* = 0.002), there was no significant relationship between ELST‐blue score and apoA2‐I Index in patients with non‐ECP.

**Conclusions:**

Patients with ECP accompanied by a high score of ELST‐blue have to be followed up carefully.

## 1 Introduction

Chronic pancreatitis (CP) is characterized by fibrosis and the reduction of pancreatic function [1]. In addition, previous studies have reported that CP was a risk factor for pancreatic cancer [2, 3], which was a fatal disease with a poor prognosis [4]. Therefore, early detection of the progression of CP is extremely important because it can also prevent the development of pancreatic cancer. New strategies have been proposed in Japan to diagnose CP in its early stages, referred to as early CP (ECP) [5, 6]. ECP is defined by clinical symptoms, abnormalities of pancreatic enzymes, dysfunction of exogenous pancreatic dysfunction, alcohol consumption, and the features of endosonography [6]. Although patients with ECP are not defined based on pancreatic fibrosis, certain populations with ECP exhibited pancreatic fibrosis predicted by severe exogenous pancreatic dysfunction. Considering early diagnosis for pancreatic elasticity or pancreatic fibrosis without pancreatic biopsies remains difficult and still under discussion [7], there is an urgent need to establish a method to clearly diagnose pancreatic elasticity in its early stages.

Despite various advances in the diagnosis of pancreatic diseases, including imaging analysis and pancreatic function evaluation, the progression of local inflammation and fibrosis remains challenging using abdominal computed tomography (CT), abdominal ultrasonography (AUS), or blood tests, even in cases of markedly impaired pancreatic function. While endosonography allows assessment of pancreatic inflammation and pancreatic fibrosis, its ability to evaluate the distribution of these pathological changes is limited [1]. In addition to various diagnostic imaging tools, pancreatic elasticity has been scored as an “elastic score” using endoscopic ultrasonography (EUS) strain elastography and used for diagnosis [7]. We have reported that the potential of EUS strain elastography combined with “Image J” software for assessing pancreatic elasticity and its relationship with pancreatic function in ECP [8]. We demonstrated that the ELST‐blue score, measured by quantified blue‐stained elastic regions on elastography images, was significantly elevated in ECP patients compared to asymptomatic patients with pancreatic enzyme abnormalities (AP‐P). Furthermore, in our previous studies, a significant correlation was identified between the ELST‐blue score and homeostatic model assessment of beta cell function (HOMA‐β) as endocrine pancreatic function of patients with ECP. [8]

Another biomarker of pancreatic cancer is apolipoprotein A2 (apoA2), which has been reported to have a characteristic processing pattern in pancreatic cancer patients [9]. In addition, we had also reported that the potential of apoA2 isoforms, particularly the lighter isoform apoA2‐AT, as a biomarker for exocrine pancreatic dysfunction [10].

In this study, we aimed to evaluate the usefulness of the ELST‐blue score to explore its potential application in identifying high‐risk groups for ECP through reflecting on pancreatic elasticity and the reduction of pancreatic function, and we tried to demonstrate whether the ELST‐blue score was significantly associated with apoA2 isoforms in patients with ECP.

## 2 Methods

### 2.1 Patients

**FIGURE 1:**
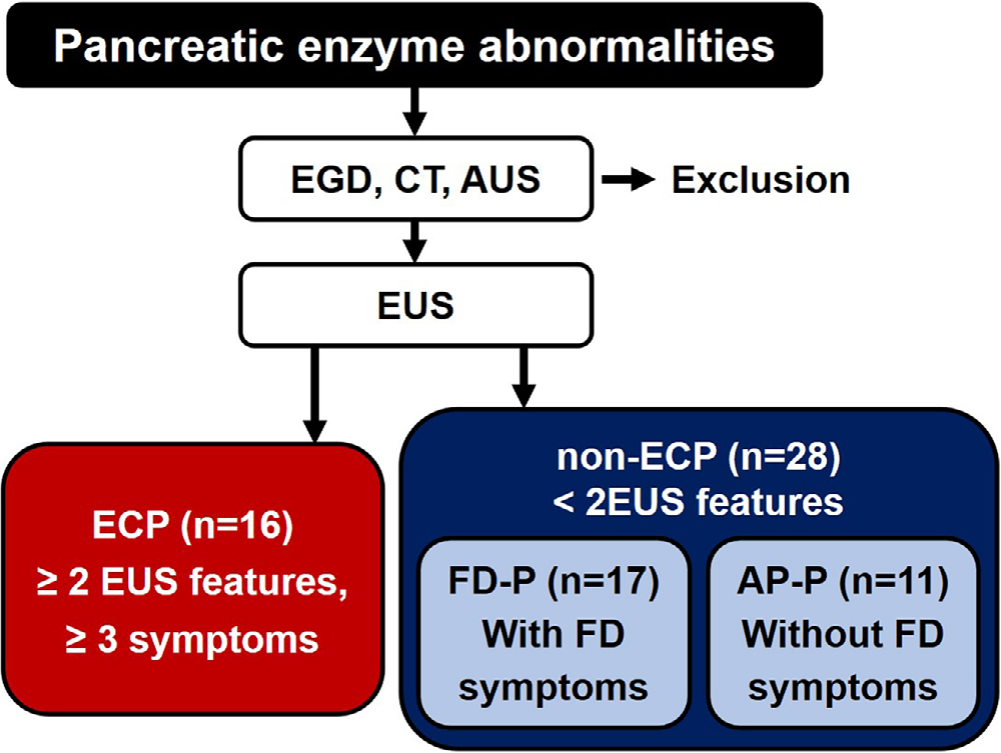
**The protocol of the study**. ECP; early chronic pancreatitis, FD; functional dyspepsia, FD‐P; functional dyspepsia with pancreatic enzyme abnormalities, AP‐P; asymptomatic patients with pancreatic enzyme abnormalities, EGD; esophagogastroduodenoscopy, CT; computed tomography, AUS; abdominal ultrasonography, EUS; endoscopic ultrasonography.

Patients with pancreatic enzyme abnormalities were enrolled in this study and underwent esophagogastroduodenoscopy (EGD), abdominal CT, and abdominal US, excluding those with liver cirrhosis, chronic heart failure, chronic kidney disease, pulmonary disease, uncontrolled diabetes mellitus, psychiatric disorders, intake of psychotropic drugs, and history of malignant disease (Figure 1). Patients were recruited from Nippon Medical School Musashi‐kosugi Hospital between April 2019 and October 2022. 44 patients with pancreatic enzyme abnormalities underwent endosonography (Figure 1). We divided two groups, one group was patients with ECP (*n* = 16) and the other group was patients with non‐ECP (*n* = 28) (Figure 1). Written informed consent was obtained from all participants, and the study was approved by the Ethics Review Committee (M‐2023‐174) of Nippon Medical School Hospital.

### 2.2 Definition of Pancreatic Enzyme Abnormalities

Serum levels of pancreatic amylase (p‐amylase), lipase, trypsin, phospholipase A2 (PLA2), and elastase‐1 were measured using the automated chemistry analyzer (AU5822 analyzer; Beckman Coulter, Brea, CA, USA). The normal ranges of pancreatic enzymes at our hospital are as follows; 18–53 (U/L) for p‐amylase, 11–53 (U/L) for lipase, 100–550 (ng/mL) for trypsin, 130–400 (ng/dL) for PLA2, and 0–300 (ng/dL) for elastase‐1.

### 2.3 Clinical Symptoms

The diagnosis of ECP needed at least three of the following: recurrent epigastric or back pain, abnormal pancreatic enzymes in blood or urine, pancreatic exocrine dysfunction, chronic alcohol intake (more than 60 g/day), and previous history of acute pancreatitis [6]. The clinical symptoms of patients were estimated based on the Rome IV criteria (score as follows: 0, none; 1, very mild; 2, mild; 3, moderate; 4, severe; and 5, very severe) [11]. Patients with functional dyspepsia (FD) had at least one of the following clinical symptoms: epigastric pain, epigastric burning, postprandial fullness, and early satiation. In this study, patients with FD were classified as FD with pancreatic enzyme abnormalities (FD‐P, *n* = 17, Figure 1) because they were also accompanied by pancreatic enzyme abnormalities, while those with non‐ECP and without FD symptoms were classified as AP‐P (*n* = 11) (Figure 1). Clinical symptoms were also evaluated using the Gastrointestinal Symptom Rating Scale (GSRS) [12]. Each item was rated according to severity on a scale of 1(no discomfort) to 7 (very severe discomfort). Information on clinical symptoms, including other above symptoms such as abdominal pain and gastroesophageal reflux, body mass index (BMI), alcohol consumption, and smoking, was collected using a questionnaire.

### 2.4 Endosonographic Assessment

An Olympus EUS system (GF‐UCT 260; Olympus, Tokyo, Japan and EU‐ME2 premier plus; Olympus, Tokyo, Japan) was used. EUS features were determined based on a previous study [6]. When there were any differences in opinions among expert endoscopists, the final diagnosis was determined by consensus following a discussion of each case on site during examination. According to the Japan Pancreatic Association [6], patients with the presence of at least two out of four EUS scores and at least three clinical symptoms were determined as ECP (*n* = 16), and other patients were non‐ECP (*n* = 28) (Figure 1). EUS score (from 0 to 4) is estimated by the sum of the EUS findings, such as hyperechoic foci or strands, lobularity, hyperechoic main pancreatic duct (MPD) margin, and dilated side branches.

### 2.5 Evaluation of the Elasticity of Pancreatic Tissue Using EUS Elastography

**FIGURE 2:**
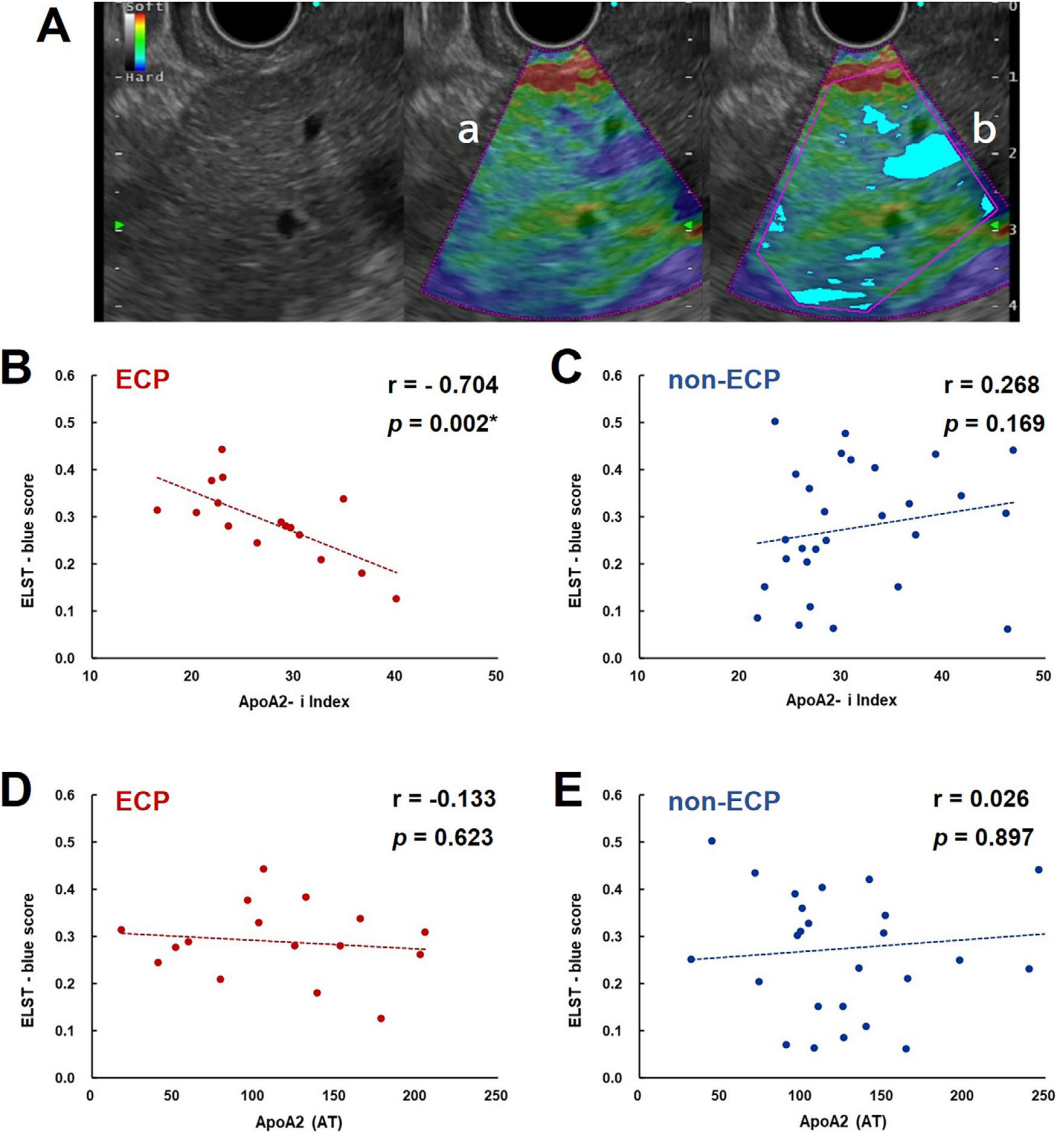
**Correlation between ELST‐blue score and apolipoprotein A2 (apoA2)‐i Index in patients with ECP and non‐ECP**. (A) Image indicates endoscopic ultrasonography (EUS) elastography for ELST‐blue score calculation. a: The measurement region, the red area means soft tissues, and the blue area indicates hard tissues. b: The light‐blue area is the region that shows blue density above a certain level. (B) The relationship between the ELST‐blue score and the apoA2‐i Index of patients with ECP. (C) The relationship between the ELST‐blue score and the apoA2‐i Index of patients with non‐ECP. (D) The relationship between ELST‐blue score and apoA2 (AT) in patients with ECP. (E) The relationship between the ELST‐blue score and apoA2 (AT) in patients with non‐ECP. ECP, early chronic pancreatitis.

EUS elastography is an imaging technique that visualizes tissue elasticity with 256 levels of color; soft areas of tissue are shown in red and hard areas are shown in blue [13] (Figure 2A). We evaluated strain elastography using the Olympus GF‐UCT 260 convex endosonographic videoscope and EU‐ME2 ultrasound processor. Strain elastography measured the amount of point variation due to pressurization in real time. The pancreatic head was measured from the duodenal bulb, and the pancreatic body and tail were measured from the gastric body using an EUS scope. Each area was measured for several seconds, and an image was automatically created from the average of several seconds of measurement data. The pancreatic head, body, and tail were each measured three times.

“ELST‐blue” was defined using the open‐source software ‘ImageJ’ developed by the National Institute of Health [8]. The measurement region was established from the EUS strain elastography (Figure 2A, a) and was maximized within the field of view, adhering to exclusions of large vessels, ducts, or cystic changes. Then, the blue areas within the region that show blue density above a certain level are marked (Figure 2A, b, light‐blue area). The threshold for the blue region was set as follows: Hue: 150–200, Saturation: 60–240, and Brightness: 60–180 by the color threshold function. For all images of all patients, the same threshold was used to determine the blue area using the ImageJ color threshold function. ELST‐blue was defined as the value of the blue areas to the measurement areas, and three areas were measured for the pancreatic body. We selected three endosonographic images, avoiding blood flow as the region of interest (ROI), and three images as the ROI were chosen at random. The maximum of the three measurements was taken as the ELST‐blue score for each patient. The ELST‐blue score was used for the reason as objective and reproductive assessment of pancreatic stiffness [8].

“Elastic score” was scored from the elastography images, as 0 for mostly green or red, 1 for a mixture of green, red, and blue, and 2 for mostly blue [7]. Three areas were each measured for each part, and the average was calculated. The summed score of each part was the total elastic score for each patient.

### 2.6 Measurement of Plasma apoA2 Isoforms

The apoA2‐i Index is a value derived from apoA2 isoforms measurements and is an indicator of the balance between apoA2‐AT and apoA2‐ATQ concentrations and a biomarker to infer apoA2‐ATQ/AT concentration [14].

### 2.7 *N*‐benzoyl‐p‐aminobenzoic Acid Test

The *N*‑benzoyl‑L‑tyrosyl‑p‑aminobenzoic acid (BT‐PABA) test is an examination to evaluate chymotrypsin activity by measuring the urinary PABA excretion rate and is used to assess the pancreatic exocrine function [15, 16].

### 2.8 Statistical Analysis

The two‐tailed unpaired *t*‐test and Pearson's chi‐squared test were used to compare continuous and categorical variables, respectively, between the two groups. The Shapiro‐Wilk test was used to determine whether or not the values followed a normal distribution. Pearson's correlation coefficient was used if the data were normally distributed, and Spearman's correlation coefficient was used if the data were non‐normally distributed. Data are presented as means and standard errors; all statistics were performed using SPSS (version 27.0; IBM, NY, USA). A *p*‐value less than 0.05 was considered significant.

## 3 Results

### 3.1 Comparison of Clinical Characteristics

No significant differences were observed between patients with ECP and non‐ECP in age, gender, and BMI (Table 1). However, the numbers of smokers and excessive drinkers were significantly higher in patients with ECP than in non‐ECP (*p* = 0.023 and *p* = 0.048, respectively) (Table 1). There were no significant differences in BT‐PABA as exocrine pancreatic function and IRI as endocrine pancreatic function between patients with ECP and non‐ECP (Table 1). There were no significant differences in GSRS between patients with ECP and non‐ECP (Table 2).

**TABLE 1:** Comparison of clinical characteristics.

	ECP (*n* = 16)	non‐ECP (*n* = 28)	*p*‐Value
Age (year)	62.5 ± 3.3	60.8 ± 3.3	0.733
Gender (Male/Female)	10 / 6	11 / 17	0.138
BMI	22.9 ± 0.9	22.1 ± 0.7	0.489
Smokers	10	7	0.023*
Excessive drinkers	2	0	0.048*
BT‐PABA (%)	54.6 ± 5.8	65.7 ± 3.8	0.105
IRI (µU/mL)	11.7 ± 2.7	11.0 ± 1.9	0.843

Data are presented as mean ± standard error.

BMI, Body Mass Index; BT‐PABA, *N*‑benzoyl‑L‑tyrosyl‑p‑aminobenzoic acid; ECP, early chronic pancreatitis; IRI, immunoreactive insulin.

Excessive drinkers are defined as those who consume more than 40 g/day of alcohol for men and 20 g/day for women.

^*^ Asterisks indicate *p*‐value of less than 0.05 for ECP compared to non‐ECP.

**TABLE 2:** Comparison of clinical symptoms between patients with ECP and non‐ECP.

	ECP (*n* = 16)	non‐ECP (*n* = 28)	*p*‐Value
Epigastric pain	3.20 ± 0.37	2.33 ± 0.29	0.074
Epigastric burning	2.07 ± 0.28	1.88 ± 0.22	0.595
Postprandial fullness	3.00 ± 0.43	3.13 ± 0.26	0.791
Early satiation	2.33 ± 0.35	3.00 ± 0.26	0.124
Global GSRS	2.51 ± 0.31	2.22 ± 0.18	0.382

	ECP (*n* = 16)	non‐ECP (*n* = 28)	*p*‐Value
Gastroesophageal reflux	2.90 ± 0.48	2.19 ± 0.22	0.196
Abdominal pain	2.44 ± 0.36	2.08 ± 0.21	0.397
Indigestion	2.65 ± 0.44	2.24 ± 0.22	0.367
Diarrhea	2.51 ± 0.36	2.14 ± 0.28	0.423
Constipation	2.07 ± 0.25	2.44 ± 0.28	0.353

Data are presented as mean ± standard error.

ECP, early chronic pancreatitis; GSRS, Gastrointestinal Symptom Rating Scale. Early satiation.

### 3.2 Pancreatic Enzyme Abnormalities between Patients with ECP and Non‐ECP

There were no significant differences in pancreatic enzyme abnormalities between patients with ECP and non‐ECP (Table 3). Similarly, there were no significant differences in serum lipid levels between the two groups (Table 3).

**TABLE 3:** Pancreatic enzyme abnormalities and serum lipid levels between patients with ECP and non‐ECP.

	ECP (*n* = 16)	non‐ECP (*n* = 28)	*p*‐Value
P‐amylase	3 (18.8)	8 (28.6)	0.496
Lipase	1 (6.3)	7 (25.0)	0.141
Trypsin	6 (37.5)	17 (60.7)	0.194
PLA2	8 (50.0)	18 (64.3)	0.484
Elastase‐1	1 (6.3)	0 (0.0)	0.161

	ECP (*n* = 16)	non‐ECP (*n* = 28)	*p*‐Value
T‐Cho (mg/dL)	218 ± 15	205 ± 6	0.449
LDL‐Cho (mg/dL)	122 ± 10	121 ± 5	0.963
HDL‐Cho (mg/dL)	59.5 ± 4.6	64.5 ± 2.8	0.328
TG (mg/dL)	136 ± 19	149 ± 24	0.729

Percentage is the ratio of patients with abnormal values. Data on pancreatic enzyme abnormalities are presented as n (%), and data on serum lipid levels are presented as mean ± standard error.

ECP, early chronic pancreatitis; HDL‐Cho, high density lipoprotein cholesterol; LDL‐Cho, low density lipoprotein cholesterol; P‐amylase, pancreatic amylase; PLA2, phospholipase A2; T‐Cho, total cholesterol; TG, triglyceride.

### 3.3 Comparison of ELST‐Blue Score and EUS Features between Patients with ECP and Non‐ECP

The ELST‐blue score was significantly higher in patients with ECP than in non‐ECP (*p* = 0.003) (Table 4A). In addition, a significant difference in elastic scores of the pancreatic body was also observed between the two groups (*p* = 0.019) (Table 4A). There was a significant difference in patients with diameter of MPD more than 2 mm (*p* = 0.031) between patients with ECP and non‐ECP, as well as in stranding or hyperechoic foci (*p* < 0.001) and lobularity (*p* = 0.040) (Table 4B). The elastic score of pancreatic tail (1.50 ± 0.26) was also significantly higher in the ECP group than in the non‐ECP group (1.07 ± 0.05), while the elastic score of pancreatic head (1.19 ± 0.10; ECP) did not differ between the two groups (1.07 ± 0.05; non‐ECP).

**TABLE 4A:** Comparison of ELST‐blue score and elastic score between patients with early chronic pancreatitis (ECP) and non‐ECP.

	ECP (*n* = 16)	non‐ECP (*n* = 28)	*p*‐Value
ELST‐blue score (body)	0.320 ± 0.024	0.218 ± 0.020	0.003*
Elastic score (head)	1.19 ± 0.10	1.07 ± 0.05	0.326
Elastic score (body)	1.44 ± 0.16	1.04 ±0.04	0.019*
Elastic score (tail)	1.50 ± 0.26	1.07 ± 0.05	0.047*

Data are presented as mean ± standard error.

ECP, early chronic pancreatitis.

^*^ Asterisks indicate *p*‐value of less than 0.05 for ECP compared to non‐ECP.

**TABLE 4B:** Comparison of endoscopic ultrasonography (EUS) features between patients with early chronic pancreatitis (ECP) and non‐ECP.

	ECP (*n* = 16)	non‐ECP (*n* = 28)	*p*‐Value
Dilater of MPD	4 (25.0)	1 (3.6)	0.031*
Lobularity	6 (37.5)	3 (10.7)	0.040*
Stranding / hyperechoic foci	10 (62.5)	3 (10.7)	<0.001*
Hyperechoic MPD	3 (18.8)	10 (35.7)	0.207
Dilated side branches	2 (12.5)	1 (3.6)	0.274

^*^Asterisks indicate *p*‐value of less than 0.05 for ECP compared to non‐ECP.

Data on EUS features are presented as *n* (%).

For dilater of MPD, the number of patients with diameter of MPD more than 2 mm.

Other items are scored by the presence (1) or absence (0).

### 3.4 Correlation between ELST‐blue Score and apoA2‐i Index in Patients with ECP and Non‐ECP

Considering that the ELST‐blue score may be associated with pancreatic elasticity and pancreatic fibrosis, we tried to investigate the correlation between the ELST‐blue score and apoA2 isoforms. An intense negative correlation was determined between ELST‐blue score and the apoA2‐i Index in patients with ECP (r = ‐0.704, *p* = 0.002, Figure 2B), highlighting an association between increased pancreatic stiffness, as measured by ELST‐blue score, and apoA2‐i Index. In contrast, no significant correlation was observed between these parameters in patients with non‐ECP (Figure 2C). In addition, although we tried to investigate whether the ELST‐blue score was associated with apoA2‐AT as exocrine pancreatic dysfunction, there was no significant association between the two factors in patients with ECP and non‐ECP (Figure 2D,E). Although the elastic score is a similar indicator of pancreatic stiffness as well as the ELST‐blue score, no significant correlation was observed between elastic score and apoA2AT and apoA2‐i Index in all head, body, and tail regions in either group (Table S1).

### 3.5 Correlation between apoA2 Isoforms and the BT‐PABA Test in ECP and Non‐ECP

**FIGURE 3:**
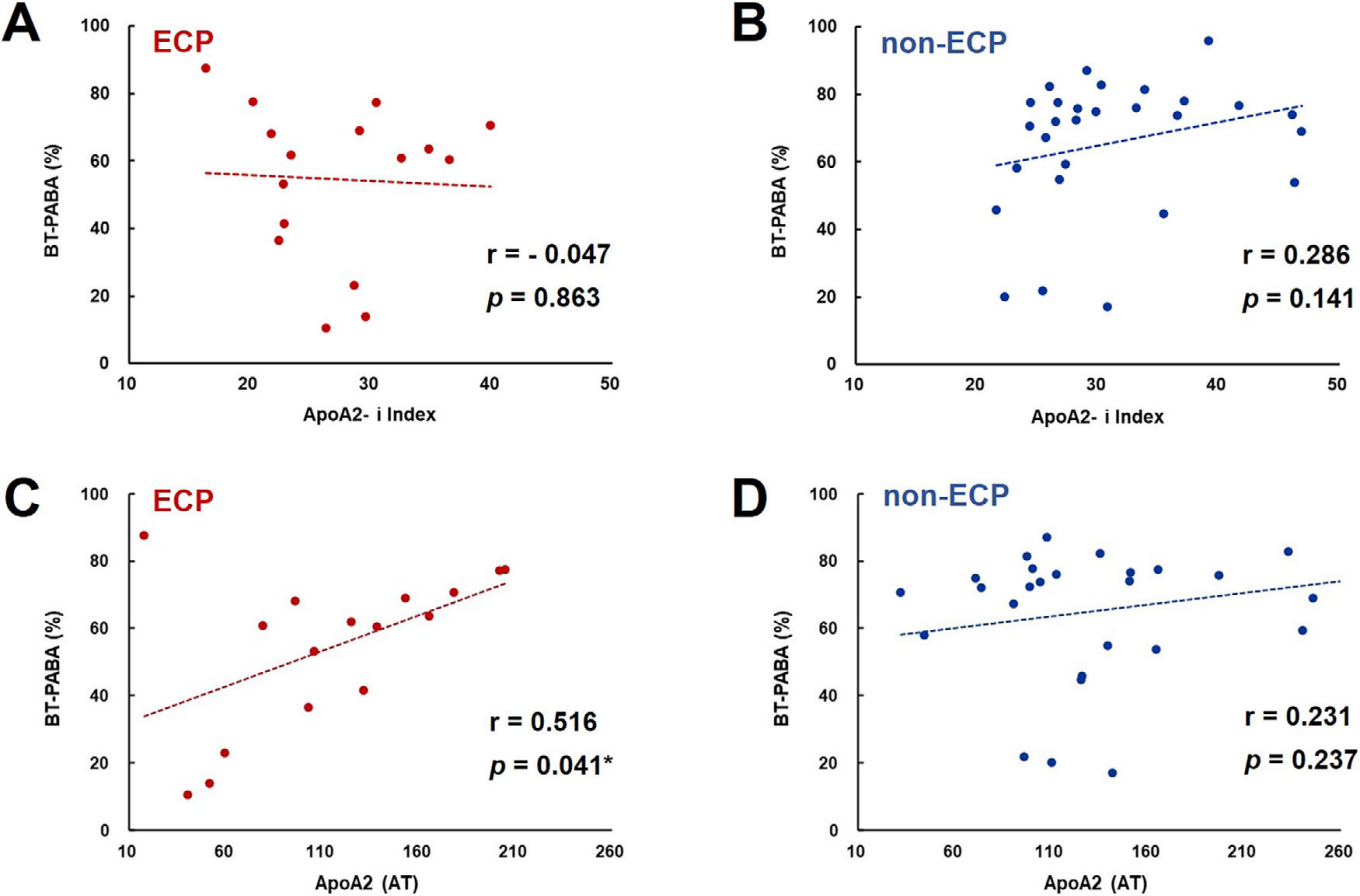
**Correlation between apolipoprotein A2 (apoA2) isoforms and the *N*‑benzoyl‑L‑tyrosyl‑p‑aminobenzoic acid (BT‐PABA) test in early chronic pancreatitis (ECP) and non‐ECP**. (A) The relationship between the BT‐PABA test and apoA2‐i Index in patients with ECP. (B) The relationship between the BT‐PABA test and apoA2‐i Index in patients with non‐ECP. (C) The relationship between the BT‐PABA test and apoA2 (AT) in patients with ECP. (D) The relationship between the BT‐PABA test and apoA2 (AT) in patients with non‐ECP.

No significant correlation was found between the BT‐PABA test and the apoA2‐i Index in either group (Figure 3A,B). A significant positive correlation was observed between the BT‐PABA test and apoA2‐AT levels in patients with ECP (*r* = 0.516, *p* = 0.041, Figure 3C), suggesting a significant link between exocrine pancreatic dysfunction and reduced apoA2 cleavage in this population. Notably, this relationship was not observed in patients with non‐ECP (Figure 3D). Furthermore, we tried to demonstrate whether the ELST‐blue score was associated with the BT‐PABA test, although there was no significant relationship between the ELST‐blue score and the BT‐PABA test in either ECP or non‐ECP (*p* = 0.857, *r* = ‐0.049 and *p* = 0.255, *r* = 0.223, respectively) (Figure S1).

## 4 Discussion

The main findings of this study are as follows: 1) a intense negative correlation was determined between ELST‐blue score and the apoA2‐i Index in patients with ECP (r = ‐0.704, *p* = 0.002), 2) epigastric pain tended to be more severe in patients with ECP (3.20 ± 0.37) than in those in non‐ECP (2.33 ± 0.29), albeit there were no significant differences in other FD symptoms or in any other items of the GSRS between two groups. 3) ELST‐blue score was significantly higher in patients with ECP than in non‐ECP (*p* = 0.003). In addition, significant difference of elastic scores in pancreatic body and tail were also observed between the two groups (*p* = 0.019 and *p* = 0.047, respectively), 4) there was a significant difference in the diameter of MPD more than 2 mm (*p* = 0.031), as well as in stranding or hyperechoic foci (*p* = 0.001) and lobularity (*p* = 0.04) between patients with ECP and non‐ECP.

Both ELST‐blue and the elastic score evaluate elasticity with pancreatic tissue using EUS elastography. While the elastic score provides an expeditious and qualitative evaluation, the ELST‐blue score offers a quantitative and objective assessment for all patients. We have also reported that the ELST‐blue score is a promising non‐invasive marker for evaluating endocrine pancreatic dysfunction in patients with ECP [8]. In this study, the ELST‐blue score had a significant negative correlation with the apoA2‐i Index, which has been reported as a biomarker of pancreatic cancer. Considering these results, we have to follow up carefully on these ECP patients accompanied by the elevation of the ELST‐blue score. Thus, in addition to endocrine dysfunction, exocrine dysfunction is a feature of CP and a risk factor for pancreatic cancer. The ELST‐blue score was not significantly (*p* = 0.857, *R* = ‐0.049) associated with the BT‐PABA test as exocrine pancreatic function in patients with ECP (Figure S1) and was also not significantly (*p* = 0.765, *R* = 0.085) associated with IRI as endocrine pancreatic function in patients with ECP (data not shown). In addition, apoA2‐i Index was not significantly associated with IRI (Figure S2) and BT‐PABA test (Figure 3). Although we speculate that the ELST‐blue score may be associated with the apoA2‐i Index through pancreatic fibrosis, further studies will be needed to clarify what the ELST‐blue score reflects in patients with ECP.

Our previous study has reported that patients with ECP and FD with pancreatic enzyme abnormalities exhibited exocrine pancreatic dysfunction [17]. In addition, Tominaga et al. have also reported that almost 70% refractory FD patients exhibited exocrine pancreatic dysfunction [18]. We have previously reported that apoA2‐AT is a useful marker for assessing exocrine pancreatic function [10]. However, the ELST‐blue score was not associated with both the apoA2‐AT or BT‐PABA test in patients with ECP in this study. Since previous studies have reported that pancreatic fibrosis and exocrine pancreatic insufficiency have been studied in CP as risk factors of pancreatic cancer [19, 20], ELST‐blue may be associated with the reduction of apoA2‐i Index and the disturbance of exocrine pancreatic function. However, in our previous study, patients with ECP with less than 35% of the BT‐PABA test were only 10% of patients with ECP [17]. We also speculated that ELST‐blue, which was reflected in pancreatic elasticity, was not correlated linearly with apoA2‐AT, which was linked to exocrine pancreatic dysfunction in patients with ECP. Further studies will be needed to clarify whether the ELST‐blue score might be associated with apoA2‐i Index and apoA2‐AT in advanced CP.

In blood, apoA2 exists as a dimer, and there are several isoforms with different carboxyl termini. The mechanism by which isoforms are altered has not been clarified, but it is likely that some pancreatic‐derived exopeptidase is involved in the blood. While apoA2‐AT and apoA2‐TQ concentrations exist in a certain balance in healthy individuals, it was found that in pancreatic cancer patients, there is a group of cases with low apoA2‐AT and high apoA2‐TQ concentrations and a group of cases with high apoA2‐AT and low apoA2‐TQ concentrations [14, 21]. ApoA2‐AT described in Figure 3C, may provide non‐invasive biomarkers for evaluating exocrine pancreatic dysfunction in patients with ECP. In contrast, apoA2‐i Index has been reported to have reduced plasma concentrations compared to healthy subjects at all stages of pancreatic cancer [22].

While our study provides novel insights into the relationship between the ELST‐blue score and the apoA2‐i Index in patients with ECP, it is important to acknowledge its limitations. First, this study focused on patients with pancreatic enzyme abnormalities, and the results may not be generalizable to the general population or to patients with other pancreatic diseases. Secondly, the sample size was relatively small, which may limit the generalizability of our findings. Thirdly, the study was conducted at a single center, which may introduce selection bias. Fourthly, we did not compare the ELST‐blue score with pancreatic elasticity through the pancreatic biopsy, which could have provided a more comprehensive assessment of the disease. Fifthly, since colour mapping may be affected by probe movement due to respiration or endoscopic manipulation, even if the use of three measurements by expert endoscopists may sometimes be insufficient to ensure reliability. Further large‐scale, multicenter, prospective studies will be needed to validate our findings and establish the clinical utility of the ELST‐blue score in the management of ECP.

Taken together, the ELST‐blue score may be a useful tool to evaluate the stage of pancreatic elasticity and is significantly associated with apoA2‐i Index as a biomarker of pancreatic cancer. Considering these results, we have to follow up carefully patients with ECP accompanied by a high score of ELST blue.

## Conflicts of Interest

The authors declare no conflicts of interest.

## Ethics Statement

The study protocol was approved by the Ethics Review Committee (M‐2023‐174) of Nippon Medical School Hospital.

## Consent

Written informed consent was obtained from all patients prior to undergoing upper GI endoscopy, endosonography, abdominal US, and CT scans to evaluate dyspeptic symptoms.

## Clinical Trial Registration

None

## Supporting information




**FIGURE S1** (A) The relationship between the ELST‐blue score and the BT‐PABA test in patients with ECP. (B) The relationship between ELST‐blue and the BT‐PABA test in patients with non‐ECP.

**FIGURE S2** (A) The relationship between apoA2‐i Index and IRI in patients with ECP. (B) The relationship between apoA2‐i Index and IRI in patients with non‐ECP.

**TABLE S1** (A) Correlation with apoA2‐AT. (B) Correlation with apoA2‐i Index.
