# Supplementary figures and images for: High Score of ELST‐Blue in Endoscopic Ultrasonography Strain Elastography May Provide a High Risk Group of Early Chronic Pancreatitis with the Reduction of Apolipoprotein A2‐i Index
Source: DEN Open. 2025 Aug 29;6(1):e70191. doi: 10.1002/deo2.70191 (PMC12395273; doi:10.1002/deo2.70191)

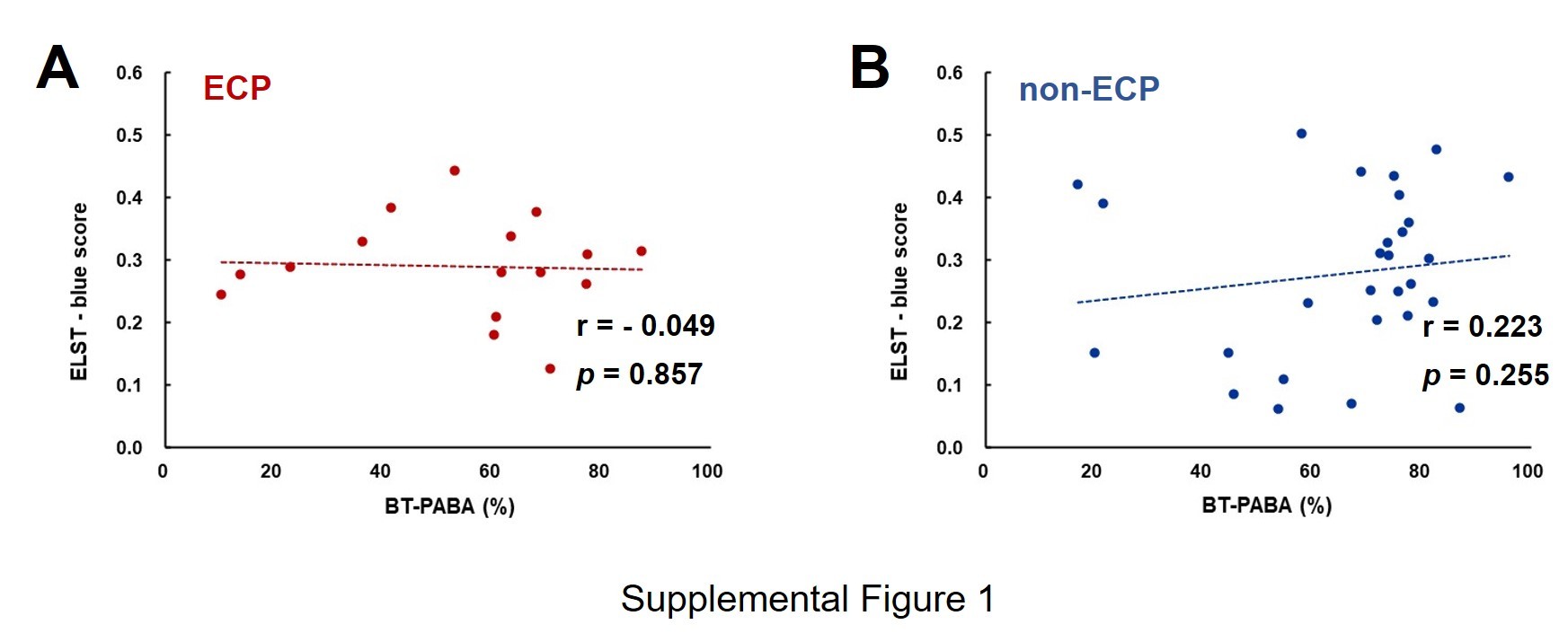

Supplement: Supplementary file 1 — FIGURE S1 (A) The relationship between the ELST‐blue score and the BT‐PABA test in patients with ECP. (B) The relationship between ELST‐blue and the BT‐PABA test in patients with non‐ECP. [file DEO2-6-e70191-s001.jpg]

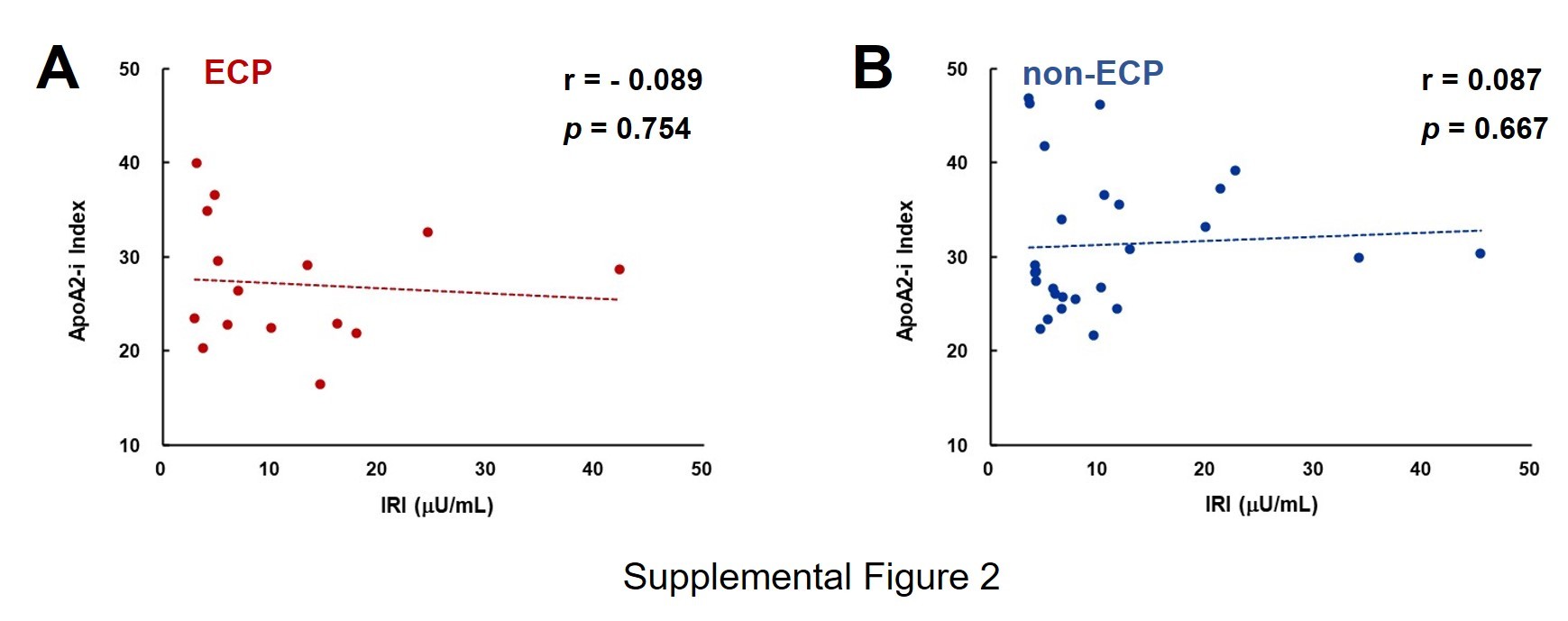

Supplement: Supplementary file 2 — FIGURE S2 (A) The relationship between apoA2‐i Index and IRI in patients with ECP. (B) The relationship between apoA2‐i Index and IRI in patients with non‐ECP. [file DEO2-6-e70191-s003.jpg]
