# Supplementary material for: High Score of ELST‐Blue in Endoscopic Ultrasonography Strain Elastography May Provide a High Risk Group of Early Chronic Pancreatitis with the Reduction of Apolipoprotein A2‐i Index
Source: DEN Open. 2025 Aug 29;6(1):e70191. doi: 10.1002/deo2.70191 (PMC12395273; doi:10.1002/deo2.70191)
Supplement: Supplementary file 3 — TABLE S1 (A) Correlation with apoA2‐AT. (B) Correlation with apoA2‐i Index. [file DEO2-6-e70191-s002.docx]

**Supplemental Table 1A.** **Correlation with apoA2-AT**

|  | ECP (n=16) | | non-ECP (n=28) | |
| --- | --- | --- | --- | --- |
|  | *r* | *p* -value | *r* | *p* -value |
| Elastic score (head) | 0.052 | 0.848 | 0.309 | 0.117 |
| Elastic score (body) | 0.056 | 0.837 | 0.101 | 0.617 |
| Elastic score (tail) | -0.278 | 0.297 | -0.073 | 0.719 |

ECP; early chronic pancreatitis. Each elastic score was not significantly associated apoA2-AT.

**Supplemental Table 1B.** **Correlation with apoA2-i Index**

|  | ECP (n=16) | | non-ECP (n=28) | |
| --- | --- | --- | --- | --- |
|  | *r* | *p* -value | *r* | *p* -value |
| Elastic score (head) | 0.052 | 0.848 | 0.363 | 0.063 |
| Elastic score (body) | 0.056 | 0.837 | 0.277 | 0.162 |
| Elastic score (tail) | 0.219 | 0.414 | 0.054 | 0.787 |

ECP; early chronic pancreatitis. Each elastic score was not significantly associated apoA2-i Index.
